# Development of the Advanced Encryption Standard

**DOI:** 10.6028/jres.126.024

**Published:** 2021-08-16

**Authors:** Miles E. Smid

**Affiliations:** 1 Formerly: Computer Security Division, National Institute of Standards and Technology, Gaithersburg, MD 20899, USA

**Keywords:** Advanced Encryption Standard (AES), consensus process, cryptography, Data Encryption Standard (DES), security requirements, SKIPJACK

## Abstract

Strong cryptographic algorithms are essential for the protection of stored and transmitted data throughout the world. This publication discusses the development of Federal Information Processing Standards Publication (FIPS) 197, which specifies a cryptographic algorithm known as the Advanced Encryption Standard (AES). The AES was the result of a cooperative multiyear effort involving the U.S. government, industry, and the academic community. Several difficult problems that had to be resolved during the standard’s development are discussed, and the eventual solutions are presented. The author writes from his viewpoint as former leader of the Security Technology Group and later as acting director of the Computer Security Division at the National Institute of Standards and Technology, where he was responsible for the AES development.

## 1 Introduction

In the late 1990s, the National Institute of Standards and Technology (NIST) was about to decide if it was going to specify a new cryptographic algorithm standard for the protection of U.S. government and commercial data. The current standard was showing signs of age and would not be up to the task of providing strong security much longer. NIST could step aside and let some other entity manage the development of new cryptographic standards, it could propose a short-term fix with a limited lifetime, or it could establish a procedure to develop a completely new algorithm. In January 1997, NIST decided to move forward with a proposal for developing an Advanced Encryption Standard (AES), which would be secure enough to last well into the next millennium. In December of 2001, after five years of effort, the finished standard was approved and published. The journey from initial concept to final standard was not straightforward. This paper covers the motivation for the development of the AES, the process that was followed, and the problems that were encountered and solved along the way. It documents a significant milestone in the history of NIST’s computer security program, which will be celebrating its 50th anniversary in 2022.

## 2 The Data Encryption Standard Reaches its Twilight

To fully understand the process for developing the AES, one must understand the issues involved with two preceding standards, the Data Encryption Standard (DES) [1] and the Escrowed Encryption Standard (EES) [2]. In the 1990s, NIST was at a crossroads. Its flagship cryptographic standard, DES, had served well, but it was approaching the end of its lifetime, and the EES, along with its required SKIPJACK algorithm [3], was not filling the void. Would NIST continue its leadership role in the development of cryptographic algorithms, or would NIST let others lead the way?

By the early 1990s, the DES had reached the height of its popularity. Not only was the algorithm being used to protect sensitive U.S. government data, it was also an American National Standards Institute (ANSI) standard known as the Data Encryption Algorithm (DEA) [4], which was used for the protection of financial information involving the transfer of funds throughout the world. In retail banking applications, DES protected the personal identification numbers (PINs) used in bankcard transactions. DES had become the *de facto* symmetric key^1^1 A symmetric key cryptographic algorithm is one that uses the same key to perform both encryption and decryption operations. standard of the U.S. commercial cryptographic product industry.^2^2 By using a U.S. government–approved cryptographic algorithm, U.S. government agencies, businesses, and industries are able to assert due diligence in the protection of their sensitive data. Both U.S. and international cryptographic products were typically built to include the DES. It is no wonder that the International Organization for Standardization (ISO) voted in 1986 to approve the DES as an international standard called DEA-1.

However, the U.S. voted against its own algorithm in ISO, and ISO later changed its mind about standardizing cryptographic algorithms, deciding that they were not an appropriate topic for standardization. In addition, the DES key size^3^3 The key size is the number of secret bits used in the DES cryptographic key. The key is a secret quantity, which adds security to the data encrypted by the algorithm. issue, which arose at the 1977 NIST Workshop on Cryptography in Support of Computer Security, had never been resolved to everyone’s satisfaction.^4^4 The workshop summary concluded that a 48 bit key would have been unacceptable, a 56 bit key was acceptable, but a 64 bit key would have been better. The 56 bit key was retained, and in the following years, Martin Hellman and Whitfield Diffie continued to challenge this choice. See Branstad D, Gait J, Katzke S (1977) Report of the Workshop on Cryptography in Support of Computer Security (National Bureau of Standards [NBS], Gaithersburg, MD), NBS Interagency or Internal Report (IR) 77-1291. https://doi.org/10.6028/NBS.IR.77-1291 In 1986, the U.S. government proposed the SKIPJACK algorithm for key escrow purposes. Given that SKIPJACK used an 80 bit key, was the 56 bit key used by the DES still providing adequate security?

### 2.1 Exhausting the DES

The first known recovery of a DES key by brute-force exhaustion (*i.e.*, trying to decrypt ciphertext with all successive keys until valid plaintext is recovered) was performed in 1997 as the result of a challenge contest sponsored by RSA^5^5 In this context, RSA represents the last names of the inventors of the RSA cryptographic system and the founders of RSA Laboratories, Ronald Rivest, Adi Shamir, and Leonard Adleman. Laboratories.^6^6 Any mention of commercial products or organizations is for informational purposes only. Such identification does not imply recommendation or endorsement by the National Institute of Standards and Technology, nor does it imply that the materials or equipment identified are necessarily the best available for the purpose. The key recovery process (known as DESCHALL) made use of the computing power of thousands of computers belonging to different organizations and individuals to recover a DES encrypted message in 96 days. Initially, there was some disagreement as to whether the amount of applied computing power was practical in most real-world applications. However, there was soon to be a second and a third challenge [5].

For the second challenge in 1998, the Cryptography Research, Inc., Advanced Wireless Technologies, and the Electronic Frontier Foundation designed and developed a DES cracker specifically to recover DES encryption keys [6]. The device, called Deep Crack, recovered the secret DES key in 56 hours.

In early 1999, a third challenge was won by recovering the DES key in half the time using Deep Crack in collaboration with distributed.net [7]. By then, it was feasible for an organization, or a combination of organizations, with sufficient resources to exhaust the DES key space.

### 2.2 DES Five-Year Reviews

Approximately every five years, NIST performed a formal review of the DES. As input to the review, NIST published a Federal Register announcement calling for comments from individuals and commercial organizations as well as U.S. government agencies. A commenter could recommend that the standard be withdrawn, revised, or reaffirmed for another five years. Although the DES had its critics, organizations with a large installed base tended to support reaffirmation unless a major weakness could be clearly identified. The DES was reaffirmed without change after its first two reviews.

The third DES five-year review occurred in 1993. It also reaffirmed the DES for another five years, but the following statement was included in the announcement.

“At the next review (1998), the algorithm specified in this standard will be over twenty years old. NIST will consider alternatives which offer a higher level of security. One of these alternatives may be proposed as a replacement standard at the 1998 review” [8].

NIST was hoping to get another five years out of DES, but a replacement was inevitable. By the fourth five-year review of the DES in October 1999, NIST qualified the use of a single DES^7^7 For each single DES block of plaintext, only one pass through the DES algorithm is used in the formation of each ciphertext block. for data encryption as follows.

“NIST can no longer support the use of single DES for many applications” [9].

NIST went on to say,

“Government agencies with legacy single DES systems are encouraged to transition to Triple DES. Agencies are advised to implement Triple DES when building new systems” [9].

Triple DES uses two or three independently generated DES keys to encrypt a block of plaintext by performing three DES operations: an encrypt operation followed by a decrypt operation followed by a final encrypt operation. The security is improved over a single application of DES,^8^8 The two-key version provided about 80 bits of security, whereas the three-key version provided about 112 bits of security. but the time (or computations) required to encrypt is increased by approximately a factor of three.

Nevertheless, DES continued to be approved for legacy applications until May 19, 2005, when its withdrawal was finally announced in the Federal Register [10].

## 3 Escrowed Encryption

Since the development of the DES, there have been concerns expressed by some U.S. government agencies that strong, unbreakable encryption techniques could jeopardize certain law enforcement and national security interests. Traditionally, under certain specific conditions—*e.g.*, a court order from a judge based on probable cause—members of the U.S. Federal Bureau of Investigation (FBI) could be granted a court order permitting the FBI to tap the phones of suspected criminals. However, in the world of data communications, if the criminals enciphered their communications with an encryption algorithm such as the DES, the task of recovering the communications would be significantly more difficult.

So on April 16, 1993, the U.S. government announced a new encryption initiative consisting of a classified encryption algorithm, SKIPJACK, that would be implemented in a special tamper-resistant device called the Clipper Chip and a key escrow system that would permit U.S. government agents to recover the corresponding plaintext from intercepted ciphertex [11]. The hardware implementation of SKIPJACK and the tamper-detection features were intended to protect against the subversion of the key escrow system. As in the case of telephone wiretaps, the U.S. government agents were required to obtain a court order based on probable cause. Later in 1994, NIST produced the EES, Federal Information Processing Standards Publication (FIPS) 185, which described a basic mechanism for recovering the SKIPJACK key from the transmitted ciphertext. A detailed description of how the key escrow system worked can be found in IEEE Communications Magazine [12]. Some in the U.S. government may have regarded the key escrow system and the SKIPJACK encryption algorithm to be the eventual replacement for the aging DES algorithm.^9^9 The key escrow system was not the first program considered as a possible replacement for the DES. In the 1980s, the U.S. National Security Agency (NSA) launched its Commercial COMSEC Endorsement Program (CCEP), which was intended to use only secret cryptographic algorithms designed by NSA and implemented only in hardware. See Diffie W, Landau S (2007) Privacy on the Line: The Politics of Wiretapping and Encryption (MIT Press, Cambridge, MA), pp 72–73. Available at https://mitpress.mit.edu/books/privacy-line-updated-and-expanded-edition.

Almost immediately, there were concerns in the academic community. While the DES was publicly defined and could be publicly evaluated, the classified SKIPJACK algorithm was not. The U.S. government tried to address this concern by selecting a panel of five experts (Ernest Brickell, Dorothy Denning, Stephen Kent, David Maher, and Walter Tuchman) and asking them to analyze the security of the algorithm. An Interim Report was produced, which concluded that “There is no significant risk that SKIPJACK *c*an be broken through a shortcut method of attack” [13]. Nevertheless, few minds were changed about the overall process. Some argued that just having the escrow feature weakened the security of the encryption system.

Many builders of commercial cryptography products had concerns that if they implemented key escrow in their products, those products would likely not be purchased outside of the United States. U.S. companies wanted to make products that could be sold worldwide, and they did not want to have special variations for different countries.

In 1994, the key escrow system suffered a significant blow when an AT&T Bell Laboratories researcher, Matt Blaze, discovered a security failure in the key escrow protocol itself [14]. Might this flaw have been avoided if a larger community had been involved in the design of the key escrow protocol?

NSA implemented SKIPJACK in its Fortezza PCMCIA^10^10 Personal Computer Memory Card International Association. cards, but, in general, the demand for EES products remained limited. By 2001, key escrow was virtually abandoned [15]. The SKIPJACK algorithm was made public in 1998. At that time, it was considered to be a strong 80 bit key algorithm. However, by 2015, SKIPJACK was disallowed by NIST for encryption because 80 bit keys no longer afforded adequate security.

In spite of the negative publicity regarding the U.S. government’s key escrow system, the concept of key recovery in certain situations proved to be a reasonable one. For example, a corporation whose employees regularly encrypt company proprietary data with user keys may want to be able to recover the plaintext data in the event that the key or keys are forgotten, lost, or withheld. Therefore, the company may want a cryptographic system that permits the recovery of such keys without involving the employee. During the years of the NIST key escrow program, several vendors incorporated key recovery capabilities into their systems so that the companies owning the data could recover the data without input from the employee that performed the encryption. These systems provided the recovery capability to the system owner rather than to the U.S. government, but the U.S. government could still request the data from the owners using the previously defined legal processes for obtaining unencrypted data.

## 4 Time for a New Standard

With the DES in its twilight and SKIPJACK under the key escrow cloud, it was clear that NIST needed a new symmetric block cipher. Without such a block cipher, several of NIST’s standards for data encryption and authentication would be useless. U.S. government agencies, product builders, and product users who needed strong encryption and supported U.S. government standards would be left without recourse.

NIST could upgrade its encryption standard to Triple DES. This more secure, but slower, algorithm would likely suffice for a number of years, but it was unclear how the cryptographic community and prospective users would view using an algorithm for which the basic engine could be exhausted. Additionally, NIST would hardly be showing leadership by merely extending the life of the twenty-year-old DES.

The alternative was to find an entirely new cryptographic block cipher to be the NIST standard. However, this alternative was not without its own problems. In the charged atmosphere of the U.S. government’s key escrow program, it appeared as though NIST was not on the side of the commercial product vendors who wanted to sell their products internationally. This was particularly awkward since NIST was an institute in the U.S. Department of Commerce, the mission of which is, in part, to promote U.S. competitiveness in the global marketplace by strengthening and safeguarding the nation’s economic infrastructure.

Some even questioned NIST’s role in developing cryptographic algorithm standards. At that time, the NIST employees involved in cryptography comprised a small, dedicated, group. The design and evaluation of cryptographic algorithms were not considered significant NIST functions. The Internet Engineering Task Force (IETF) began forming its own standards development committees, and some may have preferred NIST allow the commercial sector to develop its own encryption standards.

In the past, the U.S. government would propose a single algorithm as a U.S. government standard and then hope that vendors would build conforming products. It was also hoped that U.S. government agencies and the commercial sector would both use these products. However, NIST had no authority to force the usage of its standards. For many years, U.S. government agencies could waive the use of NIST standards. When first developed, the DES offered significantly better protection than most other algorithms for sensitive unclassified data. Yet, NIST had no power to enforce its use, and several U.S. government agencies were slow implement it.

## 5 A New Process for Developing Cryptographic Standards

In the atmosphere of the late 1990s, it was clear that in order to develop a successful new encryption standard, the U.S. government would have to allow major input from organizations and individuals outside of the U.S. government.^11^11 Note that while this was a new process for the development of NIST cryptographic standards, it is the consensus process by which standards are typically developed at NIST, as well as by ANSI, ISO, and other standards developing organizations (SDOs). The Society for Standards Professionals defines consensus standards as “standards developed through the cooperation of all parties who have an interest in participating in the development and/or use of the standards. Consensus requires that all views and objections be considered, and that an effort be made toward their resolution” (SES [2019] Library—Frequently Asked Questions. Available at https://www.ses-standards.org/page/StdsFAQs). The success of the AES effort provided evidence as to the value of consensus, especially in the development of cryptographic standards. NIST’s current principles of openness, balance, transparency, integrity, and technical merit when developing cryptographic standards can be found in NISTIR 7977, Cryptographic Technology Group (2016) NIST Cryptographic Standards and Guidelines Development Process (National Institute of Standards and Technology, Gaithersburg, MD), NIST Interagency or Internal Report (IR) 7977. https://doi.org/10.6028/NIST.IR.7977 The key escrow program demonstrated that an algorithm designed, evaluated, and proposed as a standard by the U.S. government would likely have a difficult time achieving consensus. To succeed with the new standard, NIST would have to think and act differently than it did with the cryptographic standards programs of the past. A NIST AES Selection Team was formed.

### 5.1 Partnering with the International Cryptographic Community

NIST decided that the cryptographic community would have to be involved from the beginning in the development of the new cryptographic standard. This involvement would actually be a partnership. Rather than the U.S. government or a single vendor producing the algorithm, the international cryptographic community would contribute to the design, evaluation, and selection of the new standard. NIST hoped that such a partnership would lead to acceptance of the final selection. Exactly how this would work was yet to be determined, but the expertise of the academic community in the study of cryptographic algorithms had grown tremendously since the early days of the DES.^12^12 For a brief discussion of the status of the public cryptographic community in 1977, see page 33 of Branstad D, Gait J, Katzke S (1977) Report of the Workshop on Cryptography in Support of Computer Security (National Bureau of Standards, Gaithersburg, MD), NBSIR 77-1291. https://doi.org/10.6028/NBS.IR.77-1291 Colleges were offering courses and developing degree programs in the study of cryptography. Conferences dealing with cryptography such as CRYPTO and Fast Software Encryption were held annually, and many vendors were producing commercial cryptographic products worldwide. Surely, these talents and capabilities could help to produce a better and more acceptable standard.

However, such a partnership could be problematic. Both sides would have to give up something in order to get something. Could the U.S. government give up some of the controls that it used in the past? Would the cryptographic community be willing to contribute its expertise to develop a U.S. government standard? Cooperation between the U.S. government and the international cryptographic community in an open process to produce a commercial cryptographic standard had not been done before. Would it work?

### 5.2 Initial Concerns

Some problems with the partnership idea were apparent from the beginning.

(1) **National Security:** NSA’s comments carried a great deal of weight with NIST. Would NSA decide not to participate in this cooperative program? Even worse, would NSA object to the entire effort? One of the initial tasks was to query NSA about its thoughts on this new approach. Fortunately, there was no objection, and NIST proceeded.
(2) **Key Size:** From the very beginning of its lifetime, the DES key size was under suspicion, and this concern never went away. In the beginning, NIST would argue that its key size was adequate for its intended purpose, but as time went on, the increases in computing power took their toll. The DES, with its fixed-length key size, became only weaker as computational efficiency increased. NIST did not want to have to fight key-size battles again with the AES. How could a key-size controversy be avoided? At what size will no one argue that it should be larger, and at what size will no one argue that the size is excessive (*i.e.*, where an unnecessarily large key size seriously hampers the efficiency and performance of real-world implementations)?
(3) **Export Control:** Even though the DES was a public algorithm, the export of DES products from the United States was strictly controlled. In fact, the U.S. government required that the effective key size of exported products had to be reduced from 56 bits to no more than 40 bits. This reduction of the key size was very unpopular with U.S. vendors, who wanted to sell products overseas. Could the AES have such export controls if the international cryptographic community participated in its design and evaluation?
(4) **Participation of the Cryptographic Community:** Clearly, NIST would be asking for significant contributions of time and effort from the cryptographic community. However, the AES Selection Team did not have funds to pay for external cryptographic research, and even if it did have some funding, the work would likely be awarded only to a single vendor, thus leaving all other parties out of the process. A much better approach seemed to be running a contest whereby candidate algorithms could be submitted for consideration. In addition, the cryptographic community could be invited to evaluate and comment on the candidate algorithms. To the winning candidate, NIST would offer the honor of being selected as a U.S. government–approved cryptographic algorithm, and to the evaluators, NIST would offer interesting topics for their research papers and dissertations, thereby driving advances in the field of cryptography. It was not at all clear that the rewards of fame, honor, and published papers rather than dollars would be sufficient to provide enough candidate algorithms and to stimulate enough research for the process to work. There would be some objections, so NIST would need to encourage participation.
(5) **Algorithm Requirements:** A cryptographic algorithm is designed to meet specific requirements (*e.g.*, application, type of cryptographic algorithm, key size, block size, efficiency). Before a candidate algorithm could be developed, these requirements needed to be specified.
(6) **Rules of the Competition:** The rules of the competition would need to be fair and specified in advance. These rules would include the process and deadlines, the specification of a valid submission, and the algorithm evaluation criteria. Nothing could be worse than a competition that was perceived as unfair, ambiguous, or poorly designed.

## 6 The First Federal Register Announcement

On January 2, 1997, NIST announced in the Federal Register [16] its intention to initiate the development of a new standard (AES) incorporating an “unclassified, publicly disclosed” encryption algorithm. The announcement proposed “draft minimum acceptability requirements” and “draft criteria to evaluate candidate algorithms for comment.” NIST also announced an open public workshop on these requirements as well as on submission requirements. The AES algorithm was to be capable of protecting sensitive U.S. government information “well into the next century.” In a radical change from the past, one of the requirements mandated that “AES shall be designed so that the key length may be increased as needed.” Yet another requirement specified that “AES shall be implementable in both hardware and software.” This requirement was intended to avoid the severe mandate initially placed on both the DES and SKIPJACK that they be implemented only in hardware for U.S. government approval. However, the requirement also had significant implications on the evaluation criteria, *i.e.*, to evaluate both hardware and software efficiency. The submission package would require a complete “written specification of the algorithm” including “a software implementation and source code” as well as “an analysis of the algorithm with respect to known attacks.” It was clear that NIST intended to develop a powerful new algorithm using a radically different process, but it was still not clear that this process would work.

## 7 AES Evaluation Criteria/Submission Requirements Workshop (April 15, 1997)

Seventy-three participants attended the workshop at NIST in Gaithersburg, Maryland. NIST began by announcing its initial goals for the AES algorithm, including that it should:

(1) be a strong block cipher that would support commonly used modes of operation;
(2) be selected in a fair and open manner;
(3) be usable by both industry and the U.S. government worldwide;
(4) have a variable key size so that security could be increased when needed;
(5) be at least as secure as Triple DES; and
(6) be significantly more efficient than Triple DES.^13^13 NIST’s thinking was that if no candidate was more secure and more efficient than Triple DES, then maybe Triple DES should be chosen.

The following points were made at the workshop.

(1) Key sizes of 128 and 256 bits should be mandatory.^14^14 This was a very significant result, because the key size was openly selected rather than selected by the U.S. government.
(2) Mandatory block sizes of 128 and 256 bits were discussed, while other sizes could be optional.^15^15 Block size was important because the 64 bit block size of the DES was beginning to be vulnerable to certain cryptographic attacks.
(3) Key setup time would be important when considering the efficiency and the agility of the algorithm.
(4) Minor adjustments of candidate algorithms may be allowed, but major redesigns should not be permitted.
(5) Some participants wanted to allow for proprietary, optimized software implementations.^16^16 To meet the goal of transparency, it seemed best to deny proprietary implementations in the candidate submission packages for evaluation. However, vendors could develop proprietary implementations for their customers who required faster implementations.

Although the workshop had fruitful discussions, with some exceptions, it was not well attended by the academic cryptographic community. NIST decided that rather than holding future workshops on the NIST campus, NIST would host them in concert with existing cryptography conferences that were well attended by the international cryptographic community of organizations, academics, and independent researchers. This turned out to be a wise decision because the interests and travel budgets of cryptographers did not normally extend to U.S. government workshops. Future conferences would be held in conjunction (immediately before or after) the Fast Software Encryption and/or CRYPTO conferences. This fostered cooperation and trust between NIST and academics in cryptography.

## 8 NIST Federal Register Request for Candidate Algorithm Nominations

In the September 12, 1997, issue of the Federal Register [17], NIST called for AES candidate algorithm nominations. NIST envisioned a multiyear transition process to move from the DES to the AES. The announcement included detailed requirements for the submission packages, including mathematically optimized software implementations (ANSI C and Java), and supporting documentation (including required legal statements). Three key sizes (128, 192, and 256 bits) were required, along with a block size of 128 bits. Other combinations of key and block sizes were optional. Submissions that were complete and for which the algorithms met the specified minimum acceptability requirements would be deemed to be complete and proper submissions and therefore considered as round 1 candidates. NIST intended to make the submissions publicly available (consistent with U.S. export regulations) and to request public comment and evaluation. The submissions were to be evaluated on security, cost, and algorithm implementation characteristics as specified in the request. Although NIST intended to perform its own analysis, it strongly encouraged the public to evaluate the candidates and make those results publicly available. The submission deadline was June 15, 1998.

## 9 The Schedule

The announcement requesting candidate algorithm submissions also provided information on a schedule that would lead to the selection of a winner.

(1) The evaluation process consisted of two rounds. At the beginning of round 1, NIST would host the First AES Candidate Conference and start a public comment period. At the conference, the submitters of complete and proper packages would explain and answer questions regarding their submissions. No modifications would be permitted during round 1.
(2) Approximately six months after the First AES Candidate Conference, NIST would hold a Second AES Candidate Conference. NIST would use all comments received to narrow the pool of candidates to no more than five candidates for more careful study and analysis in round 2.
(3) Before the start of round 2 evaluation, submitters would have the option to provide updated optimized implementations for use during the second round. Small modifications with written explanations could be submitted at that time. NIST would then announce the five or fewer finalists, and a round 2 evaluation period of six to nine months would then begin.
(4) Near the end of the round 2 review period, NIST would hold a Third AES Candidate Conference. Shortly thereafter, NIST would select the algorithm(s) for inclusion in the standard.

## 10 More Concerns

Given its ambitious schedule, the NIST AES Selection Team was concerned. Several potential problems threatened the success of the AES development process.

(1) **Submissions:** The team had concerns about the number and the quality of the submissions. Too many submissions could potentially overwhelm NIST and everyone else involved; too few submissions could result in none being acceptable for selection by NIST.
(2) **Analysis:** NIST had generally relied on NSA to evaluate the cryptographic strength of algorithms. Depending on the number of candidates, NIST might not have enough technical staff to cryptographically analyze each proposal. Would the comments received by NIST—along with its own analysis—be sufficient to make a good selection?
(3) **Schedule:** Generally, it takes several years to evaluate a cryptographic algorithm. The algorithm can be tested against known attacks, but often unknown attacks or previously unknown variations on known attacks sink a promising design. If the development schedule were too short, there would be concerns that NIST was making its selection based on little hard evidence. However, if the time allotted for evaluation were too long, the cryptographic community or even NIST might lose interest in the project. After much consideration, NIST picked the schedule outlined above.
(4) **Legal Issues:** NIST desired the algorithm specified in the AES to be available on a worldwide, nonexclusive, royalty-free basis. NIST could require submitters to provide information about the ownership of the algorithm and all known patents that applied to the algorithm. NIST could also require submitters to provide a statement that, if selected, the algorithm would be nonexclusive and royalty free. However, there was no apparent way for NIST to prevent a party from claiming ownership of an unknown patent upon which the algorithm infringed. The only way that this issue could be mitigated was to publish the candidate algorithms and request that NIST be notified of any known infringement. Publication would provide protection against future patents, and requests for known infringement might provide some protection against prior patents.
(5) **Export Controls:** NIST wanted to be able to export reference implementations of the AES candidates to all interested parties so that the algorithms could be evaluated. Therefore, NIST began working with the Department of Commerce’s Bureau of Export Administration. Initially, while the descriptions of the algorithms could be exported, the software reference implementations could be exported only if the recipient agreed to use the algorithms only for personal use. Later on, others outside of the United States produced reference implementations, and the issue became moot.
(6) **An NSA Submission:** The call for candidate algorithms was open to all who wished to submit. What would be the consequences if NSA submitted an algorithm? If NSA submitted a candidate, then it would be in competition with the other candidates. This could put NIST and/or NSA in an awkward position. If the NSA submission won the competition, some in the international cryptographic community might think that NIST led a sham competition. They might even think that the whole process was rigged from the very beginning to produce a U.S. government algorithm as the ultimate winner. On the other hand, if the NSA submission lost the competition, NSA staff might be angry and embarrassed. In the end, NSA chose not to submit a candidate algorithm.
(7) **NSA Analysis:** While NIST would want the benefit of the NSA staff’s expertise in cryptographic analysis, a difficult question arose as to what NIST would do if NSA informed NIST that it found a classified weakness in one of the candidate algorithms. NIST could be put in a position where it would have to deprecate a submission without making the reason public. Clearly, NIST did not want to get into such a situation. Discussions were held with NSA on the topic, but no good solution was found. NIST decided to proceed and hope that such a situation would not arise. Ultimately, it did not.

## 11 First AES Candidate Conference

The First AES Candidate Conference was held in Ventura, California, on August 20–22, 1998, immediately preceding the CRYPTO conference in Santa Barbara, California [18]. Two-hundred members of the global cryptographic community attended. NIST hoped that the attendees of CRYPTO ’98 would come to Ventura early in order to attend the candidate conference. This hope was definitely realized.

NIST began by reporting on the results of its Federal Register call for candidate algorithms. NIST had received 21 packages, but six were incomplete. That left 15 candidates from 10 different countries (see Table 1). NIST was very pleased with the number and the diversity of the candidates.

**Table 1:** AES round 1 candidate algorithms, with submitters and countries of origin.

Algorithm	Submitters	Countries
CAST-256	Entrust Technologies, Inc.	Canada
CRYPTON	Future Systems, Inc.	Korea
DEAL	Richard Outerbridge, Lars Knudsen	Canada
DFC	Centre National pour la Recherche Scientifique (CNRS)	France
E2	Nippon Telegraph and Telephone Corp.	Japan
FROG	TecApro International S.A.	Costa Rica
HPC	Rich Schroeppel	United States
LOKI97	Lawrie Brown, Josef Pieprzyk, Jennifer Seberry	Australia
MAGENTA	Deutsche Telekom AG	Germany
MARS	IBM	United States
RC6	RSA Laboratories	United States
Rijndael	Joan Daemen, Vincent Rijmen	Belgium
SAFER+	Cylink Corporation	United States
Serpent	Ross Anderson, Eli Biham, Lars Knudsen	United Kingdom, Israel, Norway
Twofish	Bruce Schneier, John Kelsey, Doug Whiting, David Wagner, Chris Hall, Niels Ferguson	United States

After NIST announced the candidates, the submitters of each algorithm briefed the attendees on their submissions. Questions flowed freely from the cryptographers in the audience. Possible attacks and weaknesses associated with some algorithms were proposed and discussed in real time.

NIST was very pleased with the response to its call for proposals and the participation at the First AES Candidate Conference. In its closing remarks, a NIST AES Selection Team member stated that NIST “hopes to select a single algorithm for the AES that will have a high degree of public confidence from its inception” and that it “is proceeding carefully but relatively rapidly, so that U.S. government agencies will soon have a newer, stronger, and more efficient security technology available for protecting sensitive information for the next 30 years.”

Saying, “Let the games begin,” NIST invited all attendees to the Second AES Candidate Conference to be held in Rome, Italy, the next year in March.

## 12 Second AES Candidate Conference

On March 22–23, 1999, 180 members of the global cryptographic research community from 20 countries met in Rome immediately preceding the Fast Software Encryption Workshop to discuss the selection of five or fewer finalists of the AES competition [19]. On September 14, 1998, NIST had issued a Federal Register request for comments on the 15 round 1 candidate algorithms. Now was the time for final discussions before NIST would narrow the field. The three main evaluation factors were security, efficiency, and flexibility, but intellectual property was also a concern. Attacks had been claimed against LOKI97, FROG, MAGENTA, DEAL, and SAFER+. On the other hand, MARS, RC2, Rijndael, Twofish, E2, CAST-256, Serpent, and HPC seemed to fare better.

The idea of a security margin was introduced. It involved comparing the number of rounds used by the algorithm against the largest number of rounds for which an attack could be demonstrated [20]. If the difference were large, one might think that the algorithm had a large safety margin. If the difference were small, one might conclude that the algorithm was operating at maximum efficiency but may be insecure (if a slightly better attack on a few more rounds could be found). It was suggested that the number of rounds should perhaps be variable. Timing and power analysis attacks were also considered.

As far as computational efficiency was concerned, most of the comparisons were made on software implementations. Testing was done by NIST on its reference implementation using the optimized implementations in the submission packages. NIST also compared its results with those of Brian Gladman and those of the Twofish team. The five fastest algorithms across the three implementations were CRYPTON, MARS, RC6, Rijndael, and Twofish. Also, the three slowest algorithms over the three platforms were DEAL, LOKI97, and MAGENTA. Some of the submitters discussed implementations where they felt that their algorithms functioned more efficiently (*e.g.*, 64 bit processors). Software efficiency has many variables,^17^17 For example, software efficiency variables may include algorithm design, programming language, platform, programmer skill, key schedule, mode of encryption, and number and size of input data blocks. and NIST as well as others would continue to study the efficiency question.

Minor modifications had to be submitted by May 15, 1999, and NIST intended to announce the finalists sometime in the summer of 1999.

At the end of the Second AES Candidate Conference, the attendees responded to a feedback sheet that asked them which of the candidates should be selected as an AES finalist and which candidates should not be selected as a finalist. This gave the positive and negative votes for the 15 candidates shown in Table 2. The yes-no column indicates the “yes” votes minus the “no” votes for a given algorithm, and the rank is based on this difference. The “?” column indicates undecided votes. While this feedback might be considered to be some measure of the overall favorability of the algorithms among the responding attendees, it was considered only unofficial conference feedback by NIST.

## 13 The Finalists

In its *Status Report on the First Round of the Development of the Advanced Encryption Standard* [21], NIST reported on its analysis of the 15 candidate algorithms based on all the information available to date. NIST had received 28 papers containing comments on the candidates that were posted on the AES home page. Exactly five finalists were selected for round 2. For each finalist, no significant security vulnerabilities were discovered, and each appeared to be sufficiently efficient in software, but round 2 would subject them to further analysis. Table 3 lists each finalist and summarizes the strengths of each.

**Table 2:** Summary of attendee feedback from the Second AES Candidate Conference.

Algorithm	No Response	Yes	?	No	Yes-No	Rank
Rijndael	7	77	19	1	76	1
RC6	4	79	15	6	73	2
Twofish	9	64	28	3	61	3
MARS	5	58	35	6	52	4
Serpent	6	52	39	7	45	5
E2	11	27	53	13	14	6
CAST-256	12	16	58	18	−2	7
SAFER+	13	20	47	24	−4	8
DFC	12	22	43	27	−5	9
Crypton	14	16	43	31	−15	10
DEAL	10	1	22	71	−70	11
HPC	12	1	13	78	−77	12
MAGENTA	9	1	10	84	−83	13
Frog	11	1	6	86	−85	14 (tie)
LOKI97	10	1	7	86	−85	14 (tie)

**Table 3:** AES round 2 candidate algorithms and algorithm strengths.

Algorithm	Algorithm Strengths
MARS	Large security margin; good to excellent performance on 32 bit platforms; expandable key size.
RC6	Fast to very fast performance on 32 bit platforms; simple structure facilitating analysis; fast key setup; expandable key size; key size, block size, and round number fully parameterized.
Rijndael	Excellent performance across platforms; good security margin; well suited to smart cards due to low random access memory (RAM) and read only memory (ROM) requirements; operations defend well against attacks on smart card implementations; fast key setup; supports expanded key and block sizes; good instruction-level parallelism.
Serpent	Large security margin; well suited to smart cards due to low RAM and ROM requirements; operations defend well against timing and power attacks; bitslice implementations allow for efficient parallel computation of S-boxes.
Twofish	Large security margin; fast across platforms; low RAM and ROM requirements; supports on-the-fly subkey generation; good support for instruction-level parallelism; admits several modes of implementation allowing for space/time trade-offs.

## 14 Federal Register Call for Round 2 Candidates

On September 15, 1999, NIST issued a Federal Register Notice requesting comments on the finalist (round 2) AES candidate algorithms [22]. The purpose was to get public input on the five finalists so that NIST could then make the AES selection. By restricting the field to five finalists, NIST hoped that the evaluations of the finalists could be more focused, the security analysis could be more intensive, and the efficiency of the finalists in hardware could be examined. A third AES Candidate Conference was scheduled for New York City in April 2000.

## 15 Third AES Candidate Conference

On April 13–14, 2000, NIST held the Third AES Candidate Conference (AES3) in New York City after the Fast Software Encryption Workshop 2000; the AES conference was attended by 230 participants. NIST again stated, “It is intended that the AES will specify an unclassified, publicly disclosed encryption algorithm available royalty-free worldwide that is capable of protecting sensitive U.S. government information well into the next century.” Security, efficiency, and flexibility were still the main criteria for evaluating the candidates.

At the First and Second AES Candidate Conferences, the relative software efficiencies of the candidates were considered. Therefore, a major portion of the third AES candidate conference dealt with hardware efficiency considerations.

“The conference was organized into eight sessions. On the first day, Session 1 was devoted to Field Programmable Gate Array (FPGA)^18^18 Field programmable gate arrays (FPGAs) are semiconductor devices that are based around a matrix of configurable logic blocks (CLBs) connected via programmable interconnects. FPGAs can be reprogrammed to desired application or functionality requirements after manufacturing. This feature distinguishes FPGAs from application specific integrated circuits (ASICs), which are custom manufactured for specific design tasks (see “What is an FPGA?” at https://www.tutorialspoint.com/cryptography/feistel_block_cipher.htm). evaluations; Session 2 to platform-specific evaluations; Session 3 to survey evaluations; and Session 4 to cryptographic properties and analysis. On the second day, Session 5 was a continuation of Session 4. Session 6 was devoted to a panel and audience discussion of AES issues; Session 7 to Application Specific Integrated Circuit (ASIC) evaluations and individual algorithm testing; and Session 8 to presentations from the submitters of the five finalist algorithms, followed by audience questions and discussion” [23].

The finalists were examined and compared with respect to many features, but no clear winner was apparent. The submitters themselves tended to feel that each finalist was probably an acceptably strong algorithm.

Two additional issues were raised during the conference.

(1) The first issue was whether multiple winners should be declared. Multiple winners would have the advantage of flexibility because, for a particular application, the the most appropriate algorithm could be selected. Also, more security might be attained (at the cost of less efficiency) by multiple encryptions with different algorithms, and if one algorithm were compromised, others would already be approved to take its place. However, implementers claimed that they would have to implement all the selected winners for interoperability reasons, adding to the cost and complexity of their implementations.
(2) The second issue was whether an AES runner-up should be selected. The runner-up would be used only in the event that the winner was found to be insecure. This would save evaluation time for a new algorithm in the event of a compromise. However, many cryptographers felt that it would be better to start the selection process all over because new attacks that were not known initially might be found on the runner-up.

After much discussion, it appeared that a single AES winner was the desired choice of most participants.

In an interesting panel of finalist submitters, each panelist was asked which algorithm, other than their own, they would favor. Vincent Rijmen liked RC6; the other four panelists said Rijndael, if that algorithm was extended to 18 or more rounds.^19^19 Rijmen did not see the need for an increase in rounds, and consensus on this point was not reached at the workshop. NIST later decided not to make any changes in the number of rounds for any of the submissions. There were also concerns that the algorithm analysis process was limited. Ronald Rivest suggested that NIST should foster more analysis after the AES was established.

## 16 And the Winner Is…

During the round 2 analysis, NIST heard a rumor that in Europe some had predicted that the IBM candidate, MARS, would win the competition. The thinking was that NIST would pick an algorithm designed by an American company and that IBM would be the favorite company because IBM and NIST had worked together on the DES development. However, the AES Selection Team was determined to select the AES based on the merits of the finalists after considering all available information. If the eventual winner happened to be from another country, that would just lend credence to the fairness of the process. The round 2 comment period closed on May 15, 2000, and the cryptographic community awaited NIST’s selection.

On October 2, 2000 [24], NIST announced the selection of Rijndael [25] and produced a paper summarizing the entire AES selection process [26]. A significant factor in the selection of Rijndael was its consistently good performance over software, hardware, firmware, and smart card implementations.^20^20 The name Rijndael was formed as a combination of the names of the two developers of the algorithm, Vincent Rijmen and Joan Daemen. On February 25, 2020, the inventors were awarded the RSA Conference Award for Excellence in Mathematics. Their work on developing the algorithm that became the AES standard was cited as one of the contributing factors in their selection. See https://www.rsaconference.com/about/press-releases/rsa-conference-2020-announces-excellence-in-the-field-of-mathematics-award.

In the February 28, 2001, Federal Register, NIST requested comments on a draft AES standard [27]. NIST also again requested comments regarding any intellectual property that may be infringed by the practice of the algorithm(s) in the draft.

Finally, on December 6, 2001, NIST announced the approval of the AES as Federal Information Processing Standard (FIPS) 197 in the Federal Register [28]. The five-year process to develop the new standard was complete.

## 17 Widespread AES Use

In the late 1990s, it became very clear that the aging DES (FIPS 46) needed to be replaced. The AES met that need. Within four months of the publication of the AES, the first implementation was validated under the NIST Cryptographic Algorithm Validation Program (CAVP). Since 2001, the CAVP has validated over 5770 AES implementations.^21^21 See NIST’s searchable database of validated algorithm implementations, under the Cryptographic Algorithm Validation Program, at https://csrc.nist.gov/projects/cryptographic-algorithm-validation-program/validation-search. With the exception of the years 2014, 2016, and 2019, the number of AES validations increased every year (see Table 4). This compares favorably to the less than 700 DES validations that CAVP performed over the past 30 years. The AES has been adopted by several non–U.S. government standards groups; for example, the AES block cipher is included in ISO/IEC^22^22 International Organization for Standardization/International Electrotechnical Commission (ISO/IEC). 18033-3:2010, it is the preferred block cipher for Institute of Electrical and Electronics Engineers (IEEE) 802.11 (Wi-Fi) secure wireless networks, and it is mandatory to implement in version 1.2 of the Internet Engineering Task Force’s (IETF) Transport Layer Security (TLS) protocol. The AES has been implemented in C, C++, C#/.net, Java, Python, JavaScript, and LabVIEW programming languages. The AES is used for archive and compression, file encryption systems, disk/partition encryption, local area network security, Internet Protocol Security (IPsec), Linux kernel’s Application Programming Interface (API), Google Allo (optional), Facebook Messenger (optional), and many other applications. A VHDL^23^23 Very High Speed Integrated Circuit (VHSIC) Hardware Description Language (VHDL). implementation developed by NSA as part of the AES development is available on the NIST Computer Security Resource Center (CSRC) website.^24^24
https://csrc.nist.gov/projects/cryptographic-standards-and-guidelines/archived-crypto-projects/aes-development Intel, Advanced Micro Devices, IBM zSeries mainframes, and SPARC S3 core processors provide AES partial algorithm instructions [29].

Although it was NIST’s intent that the AES be used for the protection of sensitive but unclassified information, NSA later announced that, “The design and strength of all key lengths of the AES algorithm (i.e., 128, 192 and 256) are sufficient to protect classified information up to the SECRET level. TOP SECRET information will require use of either the 192 or 256 key lengths. The implementation of the AES in products intended to protect national security systems and/or information must be reviewed and certified by NSA prior to their acquisition and use” [30]. Therefore, the use of the AES has been extended beyond the original scope to the protection of certain classified information.

**Table 4:** CAVP validations of AES implementations, by year.

Year	Validations		Year	Validations	
2002		45	2011		360
2003		71	2012		383
2004		94	2013		421
2005		120	2014		409
2006		156	2015		570
2007		197	2016		543
2008		269	2017		728
2009		273	2018		1057
2010		274	2019		830

## 18 AES Impact Study

In 2018, NIST sponsored a formal impact study of the AES [31]. The authors surveyed potential beneficiaries of the AES program. Seventy-four responses were received from “private sector customers, public sector customers, and cryptographic module producers, developers, and integrators.” These responders provided “sufficient quantifiable information to allow for direct estimates of economic benefits.” Ninety-five additional respondents answered survey questions that could not be used to directly estimate their organizations’ economic benefits. However, information provided by all 169 respondents could be used to extrapolate the economic benefits of the entire group.

The study computed the AES benefit-to-cost ratio and the AES net present value using three different methods. Method 1 computed the benefits from the 74 survey respondents, method 2 extrapolated benefits from all 169 respondents, and method 3 extrapolated benefits from the 169 survey respondents to industry sectors with greater than ten respondents (See Table 5).

**Table 5:** Summary of AES benefit-to-cost ratio and net present value computations.

	Method 1	Method 2	Method 3
AES benefit-to-cost ratio	28.6	70.2	1976.3
AES net present value in constant 2017 dollars	$844,500,000	$8,772,000,000	$250,473,200,000

The study concluded, “Evidence supports that estimated AES benefits are clearly underestimates. The survey responses support the conclusion that NIST’s investment in the AES has been repaid many times over, with economy-wide benefits exceeding NIST’s costs by a multiple well in excess of the extrapolated benefit-to-cost ratio of roughly 2,000” [31, p. 79].

## 19 Conclusion

"The adoption of Rijndael as the AES is a major milestone in the history of cryptography."--Ronald L. Rivest, Viterbi Professor of Computer Science, Massachusetts Institute of Technology [25]

Even more important than the many applications of the AES and its economic impact may have been the process by which the AES was developed. This process brought U.S. government agencies, academia, and industry together in an international setting to develop a strong cryptographic algorithm standard. ISO moved from a position where cryptographic standards were not considered an appropriate topic for standardization to a position where multiple cryptographic algorithms are now available for selection by the international community. The atmosphere changed from mutual suspicion to productive cooperation.

The success of the AES development effort demonstrated that by maximizing inclusiveness and transparency, NIST could develop better cryptographic standards with fewer problems and less resistance than if the U.S. government tried to develop standards without the participation of interested parties. The NIST mathematicians and computer scientists who worked on the project were proud of the results. Cooperation between the U.S. government and the cryptographic community increased trust and led to more significant analysis. As a result, the AES algorithm is now used worldwide.

The AES development process has been replicated to a great extent in development of other new NIST cryptographic standards. On November 2, 2007, NIST announced the beginning of a competition for the development of a cryptographic hash algorithm [33]. Such algorithms can be used for digital signatures, message authentication codes, key derivation functions, pseudo-random functions, and in other security applications. The process to develop and select the new hash function was based on that used for the AES. NIST worked with the cryptographic community by holding workshops and conferences. During the process, new attacks on and new desirable properties for secure hash functions were discovered. On October 2, 2012, the Keccak hash was selected as the winner, and in August 2015, NIST published its third family of Secure Hash Algorithms (SHA) in FIPS 202 [34].

On August 2, 2016, NIST issued a Federal Register Request for Comments on Post-Quantum Cryptography Requirements and Evaluation Criteria [35], and in the following year, NIST received 69 candidate algorithms that met both the minimum acceptance criteria and submission requirements [36]. As of June 2021, round 3 of that effort had winnowed the candidate pool to seven finalist algorithms and eight^†^ alternates. A difference between this effort and the AES program is that NIST plans to emerge with “many good (post-quantum) choices.” However, both the AES and the Post-Quantum Cryptography programs employ transparency, U.S. government and industry cooperation, and consensus.
